# A nationwide study of the incidence rate of herb-induced liver injury in Korea

**DOI:** 10.1007/s00204-017-2007-9

**Published:** 2017-06-20

**Authors:** Jung-Hyo Cho, Dal-Seok Oh, Sang-Hoon Hong, Heung Ko, Nam-Hun Lee, Sang-Eun Park, Chang-Woo Han, Seung-Mo Kim, Young-Chul Kim, Kang-San Kim, Chang-Won Choi, Seon-My Shin, Ki-Tae Kim, Hong-Sik Choi, Jang-Hoon Lee, Jun-Young Kim, Ji-Young Kang, Dong-Soo Lee, Yo-Chan Ahn, Chang-Gue Son

**Affiliations:** 1 Hepatology Department, Daejeon Oriental Hospital of Daejeon University, 176-9 Daeheung-ro, Jung-gu, Daejeon, 34929 South Korea; 2 The K-herb Research Centre, Korea Institute of Oriental Medicine, Daejeon, South Korea; 3 Department of Internal Medicine, Dongeui Oriental Hospital of Dongeui University, Busan, South Korea; 4 Department of Internal Medicine, Oriental Hospital of Semyung University, Jecheon, South Korea; 5 Department of Internal Medicine, Cheonan Oriental Hospital of Daejeon University, Cheonan, South Korea; 6 Department of Internal Medicine, Ulsan Oriental Hospital of Dongeui University, Ulsan, South Korea; 7 School of Korean Medicine, Pusan National University, Busan, South Korea; 8 Department of Internal Medicine, Oriental Hospital of Daegu Haany University, Daegu, South Korea; 9 Department of Internal Medicine, Kyung Hee University Oriental Medical Hospital, Seoul, South Korea; 10 Department of Internal Medicine, Oriental Hospital of Wonkwang University, Iksan, South Korea; 11 Department of Internal Medicine, Oriental Hospital of Dongshin University, Suncheon, South Korea; 12 Department of Internal Medicine, Daejeon St. Mary’s Hospital of Catholic University, Daejeon, South Korea; 13 Department of Health Service Management, Daejeon University, Daejeon, South Korea

**Keywords:** Drug-induced liver injury, Liver, Alternative medicine, Epidemiology, Herb-induced liver injury, Adverse drug reaction, Incidence

## Abstract

Discrepant incidence has been reported regarding the incidence of herb-induced liver injury (HILI). To address the growing worldwide concern of HILI, we evaluated the risk of HILI in a nationwide prospective study. Between April 2013 and January 2016, 1001 inpatients (360 males and 641 females) from 10 tertiary hospitals throughout South Korea were treated with herbal drugs and had their liver enzymes periodically measured. A total of six patients met the criteria for HILI with RUCAM scores ranging from 4 to 7. All these participants were women and developed the hepatocellular type of HILI. One HILI participant met the criteria for *Hy*’s law; however, none of six cases presented clinical symptoms related to liver injury. This is the first nationwide prospective study that estimated the extent of the incidence of HILI [total: 0.60%, 95% confidence interval (CI) 0.12–1.08; women: 0.95%, 95% CI 0.19–1.68] and described its features in hospitalized participants.

**Electronic supplementary material:**

The online version of this article (doi:10.1007/s00204-017-2007-9) contains supplementary material, which is available to authorized users.

## Introduction

Drug-induced liver injury (DILI) is one of the most important adverse drug reactions (ADRs) and accounts for 13% of acute liver failure cases in the United States (Ostapowicz et al. 2002). Herbal drugs have been broadly adopted worldwide, but concerns regarding their safety have arisen. As a major cause of DILI, herbal drugs have been reported to be responsible for 24.2% of DILI cases in China (Li et al. 2007), 9% in the United States (Chalasani et al. 2008), and 11% in Spain (Andrade et al. 2005).

The clinical use of herbal medicine and traditional herbal remedies has been popular in Korea; therefore, the Korean medical environment is suitable for studying the safety of herbal products. However, there are conflicting data regarding herb-induced liver injury (HILI) in Korea. A prospective nationwide study (from May 2005 to May 2007) attributed 30.7% of 371 DILI-related hospitalizations to herbal drugs (Suk et al. 2012), whereas another study (from January 2007 to December 2008) at nine regional pharmacovigilance centers reported that only 0.5% of 567 hepatic ADR cases were caused by herbal drugs (Kwon et al. 2012), and a study based at six regional pharmacovigilance centers (from January 2007 to December 2007) presented no herbal drug-related hepatotoxicity among 52 DILI cases in Korea (Shin et al. 2009). These causality-focused studies would produce notably different results depending on differences of the studied participants.

Conversely, determining the incidence of HILI is critical to elucidate the hepatotoxicity of herbal products. The current established annual incidence of DILI is between 10 and 15 per 10,000 to 100,000 persons among participants taking prescription medications (Björnsson et al. 2013), and 6.6 per 1000 inpatients per week (Bagheri et al. 2000). For HILI, there is lack of data obtained from prospective study designs with the exception of our previous report, which observed the liver enzymes of 313 inpatients (Jeong et al. 2012).

To assess the risk of developing HILI, we conducted a prospective nationwide study involving 10 Oriental Medicine College Hospitals in South Korea and 1001 inpatients.

## Materials and methods

### Ethics approval

The study was conducted in accordance with the Declaration of Helsinki of 2013, and the protocol was approved by the Independent Ethics Committee for Human Research at 10 Oriental Medicine College Hospitals in South Korea (authorization numbers: #djomc-109, Daejeon Oriental Hospital of Daejeon University; #2013-03, Dongeui Oriental Hospital of Dongeui University; #SMOIM-01, Oriental Hospital of Semyung University; #P2013-01, Cheonan Oriental Hospital of Daejeon University; #20130819B, Ulsan Oriental Hospital of Dongeui University; #2013010, School of Korean Medicine at Pusan National University; #DHUMC-D-13005-PRO-03, Oriental Hospital of Daegu Haany University; #KOMCIRB-2013-90, Oriental Hospital of Kyung Hee University; #WKUIOMH-IRB-2013-02, Oriental Hospital of Wonkwang University and #2013-04, Oriental Hospital of Dongshin University). In addition, this study is registered at the Clinical Research Information Service in South Korea (KCT0001279). All participants could fully comprehend the protocol before participation, including the purpose of the study as well as the possible risks and side effects, and provided written informed consent. This study was monitored by the Korea Institute of Oriental Medicine (KIOM) throughout the study period.

### Study design and participants

This was a multicenter prospective study that observed the serum biomarkers for hepatic and renal function during hospitalization. The blood tests measured albumin, total bilirubin (TB), direct bilirubin (DB), aspartate transaminase (AST), alanine transaminase (ALT), gamma glutamyl transpeptidase (γ-GTP), alkaline phosphatase (ALP), lactate dehydrogenase (LDH), total cholesterol, triglyceride, blood urea nitrogen (BUN) and creatinine as well as the complete blood count (CBC), urinalysis and viral markers. These tests were regularly repeated every 14 days until the patients were discharged. If liver injury was identified, full medical examinations (including radiographic inspection) were ordered.

This study only enrolled inpatients who were administered a daily regimen of herbal drugs for their disorders. The inclusion criteria were as follows: (1) >18 years of age, (2) expected hospital stay longer than 2 weeks (at least 11 days), (3) normal liver and renal function tests upon admission, (4) not a carrier of hepatitis B virus or hepatitis C virus, and (5) the ability to understand the study purpose. Patients with the following criteria were excluded: (1) past or present symptoms of liver disease, renal disease, toxic hepatitis, and cancer; (2) expected use of conventional drugs such as antibiotics, steroids, anti-inflammatory agents, etc. (except temporary use <3 days), (3) either a history of an organ transplant or a current prescription for an immunosuppressant regimen or (4) pregnancy. Any medications for blood pressure, diabetes mellitus, hyperlipidemia and anti-coagulation were permitted if participants started their regimen more than 1 month prior to hospitalization and presented hepatic and renal function parameters in the normal range.

### Assessment of HILI

Based on the CIOMS laboratory criteria, liver injury was defined and assumed as present if either there was an increase of >2 times the upper normal limit (UNL) in the ALT or DB parameters or there was a combined increase in the AST, ALP, and TB provided that one of these values was >2 × UNL (Rockey et al. 2010). The causality was assessed using the Roussel Uclaf Causality Assessment Method (RUCAM) score, and considered as highly probable (>8), probable (6–8) or possible (3–5). Cases with RUCAM scores ≤3 were not included in HILI. In addition, all eligible participants were reassessed by an internal medicine specialist at each hospital who understood the entire study process. The types of liver injury were classified as hepatocellular (*R* ≥ 5), cholestatic (*R* ≤ 2), or mixed (2 < *R* < 5). The ratio of serum ALT to ALP was designated as the *R* value ([ALT value/ALT UNL]/[ALP value/ALP UNL]) (García-Cortés et al. 2011).

### Statistical analysis

For continuous variables, the data are expressed as the median value with a range, whereas the categorical variables are expressed as percentages using a frequency table. The incidence of HILI was also reported as percentages with confidence intervals for specific subpopulations. All statistical analyses were performed using SPSS (ver. 18.0 KO for Windows; SPSS Inc., Chicago, IL, USA).

## Results

### Participant characteristics

**Fig. 1:**
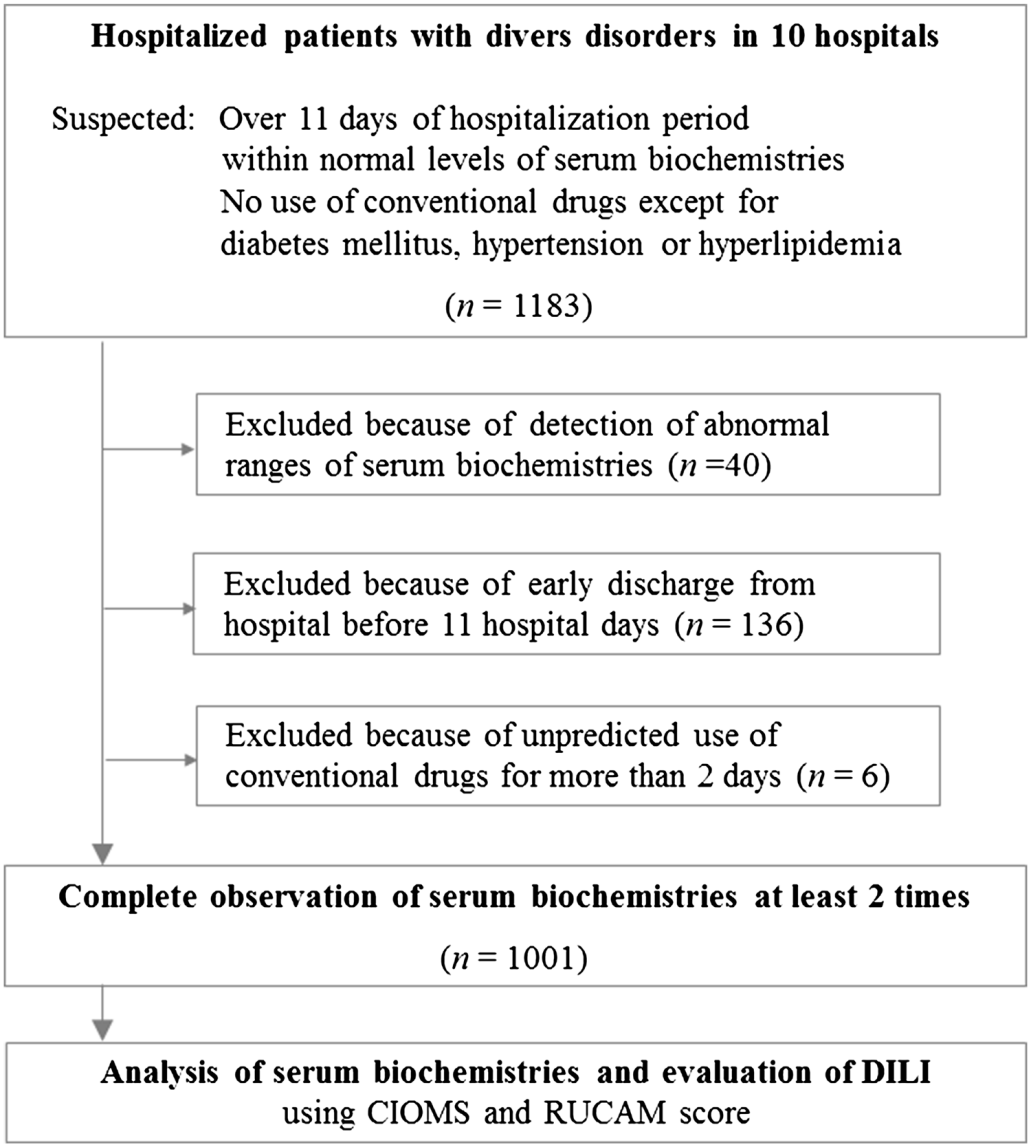
The flow of participants in the study

From April 2013 to January 2016, a total of 1183 participants (438 males and 745 females) were initially prescreened; 40 were excluded because of their abnormal serum biochemical parameter values, and 142 were dropped due to either their short hospitalization period (136 participants) or the unpredicted use of conventional drugs (6 participants). The remaining 1001 participants (360 males and 641 females) underwent at least 2 blood collections to measure the relevant biochemical serum parameters (Fig. 1). The median age of participants was 57 years old (range 18–91), and the median duration of hospitalization was 15 days (range 11–68, Table 1). The included participants had wide range of disorders, primarily in the circulatory (36.6%), nervous (20.7%), and musculoskeletal (19.0%) systems; all other systems composed the remaining 23.7%. Among the cohort, 377 participants (37.7%) took only herbal medicines, whereas the remaining 624 participants (62.3%) used a combination of herbal and conventional medicines (Table 1). A total of 440 types of traditional herbal formulae (multiple compositional herbal medicines) were prescribed to participants (data not shown).

**Table 1:** Characteristics of participants

Participant	Male/Female	Total
Number (%)	360 (36.0)/641 (64.0)	1001 (100)
Median age (year, range)	54 (18 to 91)/57 (19 to 89)	57 (18 to 91)
Median duration of hospitalization (day, range)	16 (11 to 49)/15 (11 to 68)	15 (11 to 68)
Herbal drug only (%)	137 (38.1)/240 (37.4)	377 (37.7)
Herbal and conventional drug (%)	223 (61.9)/401 (62.6)	624 (62.4)

Diagnosis by ICD-10	Number (%)	
Circulatory system (I00–I99)	366 (36.6)	
Nervous system (G00–G99)	207 (20.7)	
Musculoskeletal system and connective tissue (M00–M99)	190 (19.0)	
Ear and mastoid process (H60–H95)	36 (3.6)	
Digestive system (K00–K99)	28 (2.8)	
Respiratory system (J00–J99)	12 (1.2)	
Mental and behavioral disorders (F00–F99)	9 (0.9)	
Genitourinary system (N00–N99)	6 (0.6)	
Infectious and parasitic diseases (A00–B99)	4 (0.4)	
Endocrine, nutritional and metabolic diseases (E00–E90)	4 (0.4)	
Eye and adnexa (H00–H59)	3 (0.3)	
Blood and blood-forming organs including the immune system (D50–D89)	2 (0.2)	
Skin and subcutaneous tissue (L00–L99)	2 (0.2)	
Others	132 (13.2)	
Total	1001 (100)	

### Incidence of HILI and change of hepatic enzymes

There were six cases of HILI identified (incidence rate 0.60%, 95% CI 0.12–1.08), with 4 classified as probable (66.7%) and 2 as possible (33.3%) based on the RUCAM scores. All 6 cases occurred in females (0.95% of 641 participants, 95% CI 0.19–1.68) during a hospital stay ranging from 11 to 27 days. All HILI cases were of the hepatocellular reaction type and presented <4-fold increase over the UNL of either AST or ALT; fortunately, all the patients eventually recovered (Table 2).

**Table 2:** Characteristics of six HILI cases

Participant (sex/age)	Diagnosis	Initial detection (day)	Peak value of laboratory test*						Type of liver injury	RUCAM (score)	Follow-up
			Day	AST	ALT	ALP	γ-GTP	TB			
P1 (F/50)	Sequela of Herpes zoster	27	27	67	111	99	523	0.5	Hepatocellular	Probable (7)	Recovery
P2 (F/59)	Migraine	14	14	145	124	96	23	0.8	Hepatocellular	Probable (7)	Recovery
P3 (F/60)	Facial dystonia	19	19	37	72	141	145	0.5	Hepatocellular	Possible (4)	Recovery
P4 (F/51)	Lumbar HIVD	17	31	89	134	42	22	0.7	Hepatocellular	Possible (4)	Recovery
P5 (F/52)	Lumbar HIVD	13	23	49	86	70	34	0.6	Hepatocellular	Probable (6)	Recovery
P6 (F/59)	Thoracic strain	11	11	67	81	44	13	0.6	Hepatocellular	Probable (6)	Recovery

*HIVD* herniated intervertebral disc

* The reference ranges of laboratory tests are as follows: AST (8–35^a^ or 0–40^b^ IU/L), ALT (5–35^a^ or 0–40^b^ IU/L), ALP (30–120 IU/L), γ-GTP (0–73^a^ or 0–64^b^ IU/L), TB (0.3–1.3^a^ or 0.1–1.2^b^ mg/dL), ^a^S1 to S4, ^b^S5 and S6

Between the first and last blood test, the average of hepatic enzyme levels was increased slightly in serum AST (20.7 ± 5.7 into 21.4 ± 7.3 IU/L), ALT (19.2 ± 8.1 into 21.6 ± 11.9 IU/L), and γ-GTP (24.1 ± 16.7 into 25.1 ± 26.4 IU/L), but showed the decrease in ALP (108.3 ± 78.1 into 97.7 ± 70.3) or no change in total bilirubin (0.6 ± 0.2 mg/dL), respectively. The patterns were very similar when those parameter changes were analyzed according to gender and period of hospitalization (median hospital day ≤15 or >15 day), respectively (Supplementary Table 1). Three participants with HILI were treated with herbal medicines alone, whereas the other 3 concomitantly received herbal and conventional medicines (Supplementary Table 2).

## Discussion

It is difficult to determine the accurate incidence of DILI (including HILI) because of the lack of a gold standard for its diagnosis, underreported cases, and selection biases (Ghabril et al. 2010; Meier et al. 2005). To minimize the influences of factors other than the drugs themselves, our study only included only inpatients as a prospective design which 10 hospitals participated nationwide (including 8 of the 12 Oriental Medical Schools in the south). A total of 1001 inpatients with an average hospital stay of 15 days were enrolled in this study.

Among the 1001 participants, 6 cases of hepatic injury developed and were eventually identified as HILI based on a RUCAM score ≥3 points. These 6 cases corresponded to an incidence rate of approximately 0.60% (95% CI 0.12–1.08), which is much higher than the current established annual incidence of DILI (between 10 and 15 per 10,000 to 100,000 persons) among participants taking prescription medications (Björnsson et al. 2013), but is slightly lower than the incidence of DILI (1.4%, 95% CI 1.0–1.7) reported among 4209 inpatients from a retrospective database survey in Switzerland between 1996 and 2000 (Bagheri et al. 2000). Another prospective study conducted in France in 1997 estimated a DILI incidence of 6.6 per 1000 inpatients per week (Bagheri et al. 2000). Regarding the incidence of HILI among inpatients, compared with our results, two retrospective studies showed slightly lower or similar HILI incidences of 0.43% (5 of 1169) and 0.6% (15 of 2496) among inpatients in Korea and Japan, respectively (Woo and Kim 2015; Mantani et al. 2002).

We previously conducted a smaller prospective observational study for HILI incidence among 313 inpatients and reported a 3-fold higher HILI incidence (1.9%, 95% CI 0.4–3.4). However, that study did not limit the use of conventional medicines, and all the HILI participants were concomitantly administered herbal drugs and prescribed medications that are known to be hepatotoxic, including acetaminophen, non-steroidal anti-inflammatories, antibiotics, and steroids (Jeong et al. 2012). Recently, drug–herbal interactions have become a medical issue, including the aspects of ADRs (Rossi and Navarro 2014; Stickel and Shouval 2015). In our study, 62.3% of the participants were concomitantly administered herbal formulae and conventional drugs. Although 3 of the 6 HILI cases concomitantly took both types of medicine, these conventional drugs might not directly affect the development of HILI because inclusion criteria in our study allowed these drugs based on the normal liver function tests at the initial time point.

Of note, all the HILI cases were of the hepatocellular type and manifested only in females (0.95% of 641, 95% CI 0.19–1.68) between 50 and 60 years old. Females are generally believed to be more sensitive to DILI (Medina-Caliz et al. 2016), and it is known that women ≤60 years of age are more prone to the hepatocellular subtype of DILI (Lucena et al. 2009). None of the HILI cases presented clinical symptoms related to liver injury, and one of 6 cases meet the criteria for* Hy*’s law, which is defined as either AST or ALT ≥ 3 × UNL or TB ≥ 2 × UNL without evidence of cholestasis (ALP ≤ 2 × UNL) (García-Cortés et al. 2011). The abnormal liver enzymes in all the HILI cases completely recovered after their respective herbal drug regimens were discontinued, which indicates the importance of early screening and detection to prevent and manage DILIs, including HILI. DILI has been reported to occur most frequently within one week after taking a suspected drug (Takikawa 2010), whereas in our study, all the HILI cases were detected after approximately 2 weeks of hospitalization (median 15.5 days, range 11–27).

HILI is categorized into two subtypes: idiosyncratic, which is dose-independent and unpredictable; and intrinsic, which is dose-dependent and predictable and is primarily caused by pyrrolizidine alkaloids (PAs) (Bagheri et al. 2000). We analyzed the individual herbs that corresponded to the development of HILI, and the most commonly prescribed herb in the HILI cases was *Glycyrrhiza uralensis*; furthermore, none of administered herbs primarily consisted of PAs, including *Gynura segetum*, *Heliotropium*, *Senecio*, and *Crotalaria* (Supplementary Table 2). Accordingly, we can infer that the six cases of HILI in our study could be classified as idiosyncratic. In addition, none of the 1001 participants presented abnormalities in the serum biomarkers of renal function.

The study design significantly affected the clinical data, including the evaluation of an accurate incidence of HILI. In general, there is a tendency that the observation data from inpatients estimate a higher incidence of DILI than those from outpatients (Bagheri et al. 2000; Björnsson. 2015). The disease or hospitalization-associated stress itself also known to affect hepatic chemistries (Larrey and Faure 2011), which influenced the slight elevation in average serum levels of hepatic enzymes (AST, ALT and γ-GTP) in present study (Supplementary Table 1). As the largest prospective study for herbal drug-related safety ever conducted, this report provided a more accurate assessment of the incidence of HILI, but there are some limitations. The relatively short hospital stays could differ among those with long-term herbal use. Owing to the variety of the traditional formulae comprising multiple herbs, it is difficult to identify specific herbs with a high risk of HILI as well as the responsible mechanisms challenging. The present inpatient-based study provided well-controlled data but does not accurately represent the data for outpatients, who compose the primary population of herbal users.

In summary, we can conclude that the risk level of HILI for inpatients is approximately 0.60% and manifests predominately in women as a hepatocellular type. These data will serve as a key reference for future studies related to the safety of herbal drugs.

## Electronic supplementary material

Below is the link to the electronic supplementary material.

Supplementary material 1 (DOC 38 kb)

## Abbreviations

ADRs: Adverse drug reactions
ALT: Alanine aminotransferase
AST: Aspartate aminotransferase
CIOMS: Council for International Organizations of Medical Sciences
DILI: Drug-induced liver injury
HILI: Herb-induced liver injury
PAs: Pyrrolizidine alkaloids
RUCAM: Roussel Uclaf causality assessment model
UNL: Upper normal limit
