# Supplementary material for: A nationwide study of the incidence rate of herb-induced liver injury in Korea
Source: Arch Toxicol. 2017 Jun 20;91(12):4009–15. doi: 10.1007/s00204-017-2007-9 (PMC5719126; doi:10.1007/s00204-017-2007-9)
Supplement: Supplementary file 1 — Supplementary material 1 (DOC 38 kb) [file 204_2017_2007_MOESM1_ESM.doc]

| Supplementary table 1. Changes of hepatic enzyme values | | | | | |
| --- | --- | --- | --- | --- | --- |
| Parameters | On day of admission (top) and discharge (bottom) | | | | |
|  | AST (IU/L) | ALT (IU/L) | ALP (IU/L) | γ-GTP (IU/L) | TB (mg/dL) |
| Total  (n=1001) | 20.7±5.7  21.4±7.3 | 19.2±8.1  21.6±11.9 | 108.3±78.1  97.7±70.3 | 24.1±16.7  25.1±26.4 | 0.6±0.2  0.6±0.2 |
| Male  (n=360) | 21.0±5.7  21.4±6.8 | 21.7±8.7  24.6±12.8 | 107.6±74.2  92.1±60.2 | 30.3±19.6  29.6±19.6 | 0.7±0.2  0.7±0.2 |
| Female  (n=671) | 20.6±5.6  21.3±7.5 | 17.8±7.3  19.9±11.0 | 108.7±80.1  100.6±74.9 | 20.8±14.0  22.6±29.2 | 0.6±0.2  0.6±0.2 |
| ≤15 days (n=505) | 20.8±5.8  21.7±7.3 | 19.2±8.0  21.9±11.2 | 123.3±84.4  113.6±81.5 | 23.9±17.1  24.6±19.7 | 0.6±0.2  0.6±0.2 |
| ＞15 days (n=496) | 20.6±5.5  21.0±7.2 | 19.2±8.1  21.2±12.5 | 93.3±68.1  81.2±51.4 | 24.3±16.4  25.6±31.6 | 0.6±0.2  0.6±0.2 |
